# Physiological and Biomechanical Monitoring in American Football Players: A Scoping Review

**DOI:** 10.3390/s23073538

**Published:** 2023-03-28

**Authors:** Antonio Nocera, Agnese Sbrollini, Sofia Romagnoli, Micaela Morettini, Ennio Gambi, Laura Burattini

**Affiliations:** Department of Information Engineering, Marche Polytechnic University, Via Brecce Bianche 12, 60131 Ancona, Italy

**Keywords:** American football, bioengineering, sport, injuries, wearable sensor, portable sensor

## Abstract

American football is the sport with the highest rates of concussion injuries. Biomedical engineering applications may support athletes in monitoring their injuries, evaluating the effectiveness of their equipment, and leading industrial research in this sport. This literature review aims to report on the applications of biomedical engineering research in American football, highlighting the main trends and gaps. The review followed the PRISMA guidelines and gathered a total of 1629 records from PubMed (n = 368), Web of Science (n = 665), and Scopus (n = 596). The records were analyzed, tabulated, and clustered in topics. In total, 112 studies were selected and divided by topic in the biomechanics of concussion (n = 55), biomechanics of footwear (n = 6), biomechanics of sport-related movements (n = 6), the aerodynamics of football and catch (n = 3), injury prediction (n = 8), heat monitoring of physiological parameters (n = 8), and monitoring of the training load (n = 25). The safety of players has fueled most of the research that has led to innovations in helmet and footwear design, as well as improvements in the understanding and prevention of injuries and heat monitoring. The other important motivator for research is the improvement of performance, which has led to the monitoring of training loads and catches, and studies on the aerodynamics of football. The main gaps found in the literature were regarding the monitoring of internal loads and the innovation of shoulder pads.

## 1. Introduction

Biomedical engineering is a wide discipline that aims to use engineering methods to solve health and fitness issues. In the field of sport application, biomedical engineering may support the prevention of injuries [1] (e.g., in sports with repetitive actions like tennis [2,3] and golf [4], or in contact-sports like rugby [5,6])), may improve the safety of sports equipment (e.g., by evaluating the structure of helmets and protection [7]), and may improve technology to monitor athletes’ performance (e.g., by designing new algorithms for the analysis of cardiac signals acquired by wearable sensors) [8,9]).

American football is a sport of wide interest due to the high number of athletes involved, from youth to professionals. It is the most popular sport in the United States, with over 70,000 collegiate athletes in the last five years [10]. There is an increasing interest in American football outside of its country of origin. Indeed, there are examples in Europe, with 17 teams playing in a professional league (European League of Football) [11], in China, with 33 teams (China National Football League) [12], and in Japan, with 20 teams playing between the first and second divisions of American football (X League) [13].

This sport presents high rates of injuries [14], with a study reporting 5.5 injuries per 1000 for practice and 37.2 injuries per game for competition [14,15]. One of the typical injuries reported in American football is a concussion, with a rate of occurrence of 2.9 and 0.43 injuries per 1000 in games and practice, respectively [15]. A concussion is a form of mild traumatic brain injury, which is defined as a head trauma that impairs brain functions for a limited duration and severity [16]. Subjects with mild traumatic brain injury can experience a wide variety of symptoms, such as headache, dizziness, confusion, loss of consciousness, and amnesia [17,18]. American football athletes exposed to different brain traumas during their careers may experience mood and behavioral alterations and small cognitive impairments [19]. For these reasons, concussions are a controversial topic with significant research regarding their monitoring and prevention.

Moreover, American football participation seems to have negative consequences on cardiovascular health due to risk factors [20] or pathological features [21,22]. Despite the evidence, the reasons for the occurrences of cardiovascular issues in American football players are not properly understood [23].

Considering the many issues related to American football, biomedical engineering may be helpful in supporting the monitoring of athletes, evaluating used equipment, and leading industrial research on sports. Heart rate (HR) monitoring could be a non-invasive and simple solution to screen the internal loads of players in non-invasive and simple ways [9], aiming at preventing situations of overtraining, classifying training phases, or describing the cardiovascular fitness of the athletes [24] or describing the cardiovascular fitness of the athletes [25]. In recent years, research regarding heart rate monitoring has made significant advancements, and contactless alternatives have gained attention thanks to the opportunity to remove cumbersome wearables [26,27]. On the other hand, external loads are monitored through global positioning systems (GPS) and inertial sensors, which integrate accelerometers and gyroscopes and aim at preventing injury and enhancing performance. Recently, new wearable technologies that integrate GPS and heart rate sensors have been proposed for contact sports to prevent excessive training loads, which can cause severe fatigue and stress in athletes [28]. Other applications of worn inertial sensors relating to performance improvement include the classification of sports events and the evaluation of specific skills through kinematic description [29], such as a tennis stroke [30], a kick [31], or a tackle [32]. The possibility of instrumenting the entire team with wearables during practice and games could enable the collection of vital information for coaches to better analyze the development of a game [33]. Furthermore, biomechanical evaluations may support the prevention of injuries by monitoring physiological indexes or concussions [34,35,36,37].

Despite many papers addressing issues related to American football, no review has specifically tackled the field of biomedical engineering in this sport. Thus, the aim of this scoping review is to report on the applications of biomedical engineering research in the sport of American football, highlighting the main topics and possible challenges.

## 2. Materials and Methods

### 2.1. Information Sources and Literature Search Strategy

A literature review was conducted on three electronic databases, i.e., PubMed, Web of Science, and Scopus, following the PRISMA guidelines [38] and the extended guidelines for scoping reviews [39]. Search terms were organized into three concepts; for each of them, the following keywords with the wildcard term ‘*’ were used:

1. Population: American football, national collegiate athletic association football, college football, national football league, NCAA football, collegiate football
2. Bioengineering applications: mech*, biomech*, monitor*, screen*, analysis, eval*, pred*, Global Position System, GPS, sensor*, wearable*, track*
3. Outcomes of interest: fitness, card*, load*, kinetic*, kinematic*, motion*, performance*, fatigue, recovery, safety, workload, velocit*, acceleration*, speed, movement*, heart rate, heart rate variability (HRV), sympathetic, parasympathetic, vagal.

Search terms within each concept were combined with the Boolean operator ‘OR’ and then combined with the Boolean operator ‘AND’. The first concept related to the population was limited to the title of the papers, whereas the other two concepts were limited to the title and abstract for greater inclusion. The English language was used as a limit to filter the documents. The literature was screened from 1 January 1995 to 20 July 2022.

### 2.2. Exclusion Criteria and Selection Process

The studies were imported into the Mendeley Reference Manager system for duplicate removal. Only research papers (review papers and the proceeding were excluded) with titles, abstracts, and full text were considered. The following exclusion criteria were applied to screen titles, abstracts, and full text:

- Population, including retired players;
- Population—age lower than 18 years;
- Population, including sports other than American football;
- Content related to sports finance, sports economics, and sports organizations;
- Content related to diet, nutrition, drug usage, anthropometry, emergency, intervention, prevalence investigation, return to play, serum biomarkers, and cognitive tests.
- Content related to strength training, plyometrics training, and/or track and field exercises.

### 2.3. Data Collection Process and Synthesis Method

The objectives, study designs, sample sizes, and outcomes were collected for each paper. Considering the content of the papers, they were divided according to their specific topics into the following categories and sub-categories:

1. Biomechanics of concussion, which is subdivided into:
  (a) Laboratory reconstruction (LAB);
  (b) Monitoring with head impact telemetry system (HIT);
  (c) Wearable-sensor monitoring (WSM);
  (d) Computer modeling (CM);
2. Biomechanics of foot-wearing, which is subdivided into:
  (a) Field–footwear interactions (FFI);
  (b) Footwear bending stiffness (FBS);
3. Biomechanics of sport-related movements (SM);
4. Aerodynamics of football and catch (AFC);
5. Injury prediction (IP);
6. Heat monitoring of physiological parameters (HM);
7. Monitoring of the training load (TL).

### 2.4. Study Risk of Bias Assessment

The studies were divided into one of the following categories: sports science [40], case series [41], case-control [42], case report [43], longitudinal prospective cohort [42], before–after cohort with no control [44], descriptive laboratory design [45], quasi-experimental [46], randomized control trials [46], prediction models [47], and simulations [48]. The studies were appraised with a corresponding adequate quality of appraisal checklist.

## 3. Results

### 3.1. Study Selection

**Figure 1:**
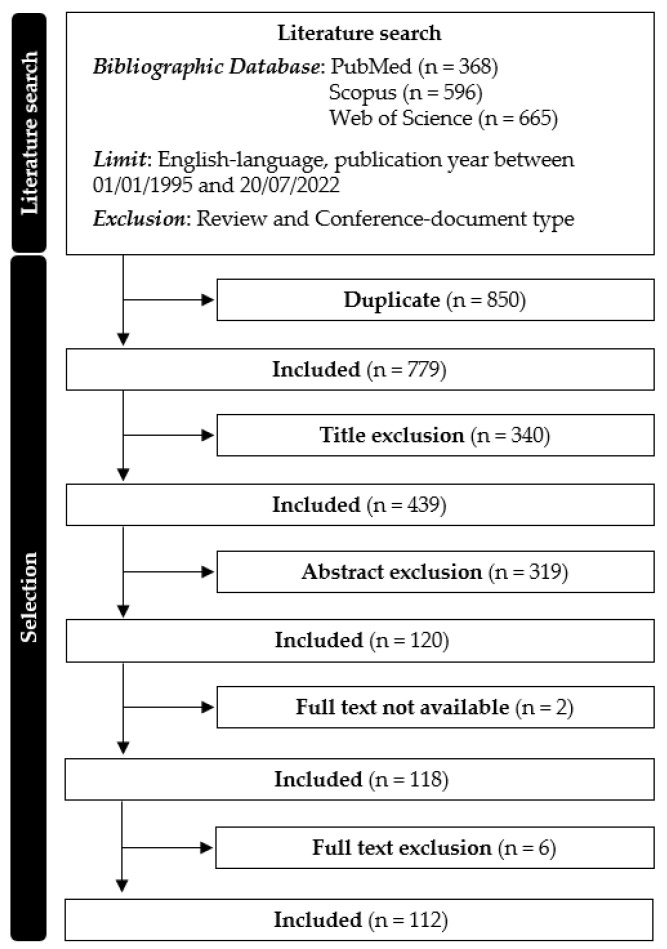
Flow chart describing the selection process (modified from [38]).

Globally, 1629 studies were identified from PubMed (n = 368), Scopus (n = 596), and Web of Science (n = 665). After duplicate removal, the title screening procedure reduced the studies to 439; the abstract and full-text screening reduced the studies to 112. The selection procedure is summarized in Figure 1.

**Figure 2:**
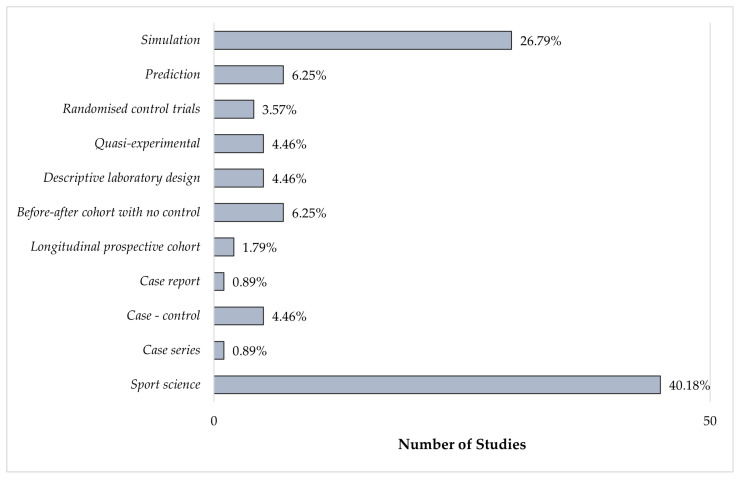
Bar chart of the study design analysis.

**Figure 3:**
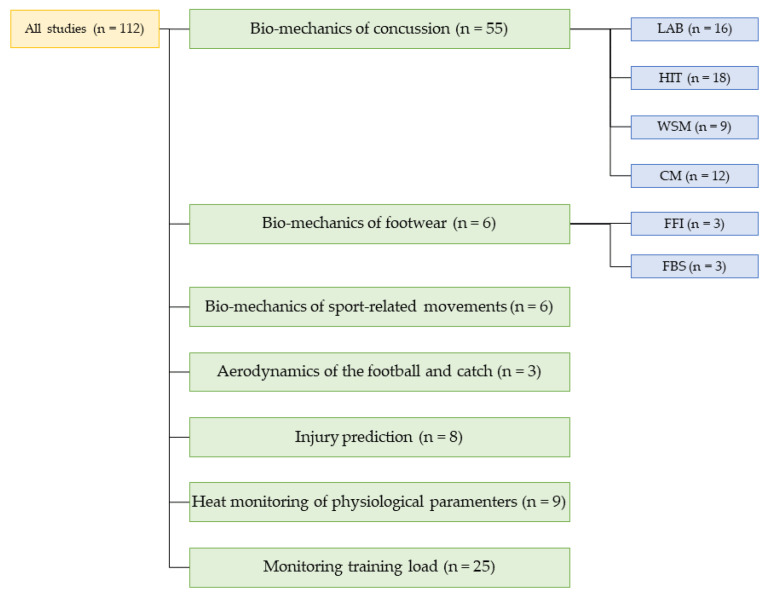
Tree chart of the distribution of studies into categories and sub-categories.

**Figure 4:**
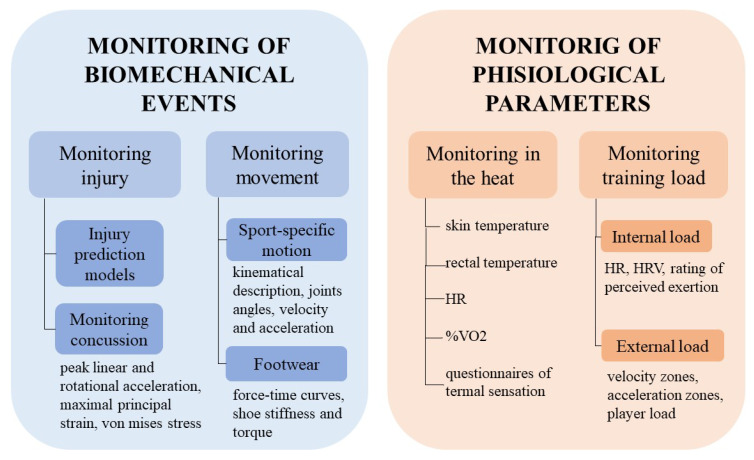
Summary of the topics and the typical parameters used for each topic.

**Figure 5:**
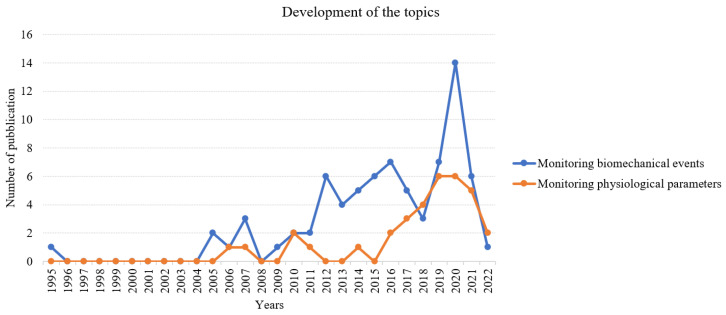
Development of the topics through the years.

The bar chart of the study design analysis is reported in Figure 2. The tree chart of the distribution of the studies into categories and sub-categories is reported in Figure 3. Given the great amount of analyzed literature, the topics can be clustered into two macro-categories to facilitate the reading in Figure 4. The first one is the monitoring of biomechanical events; it contains the biomechanics of concussions and footwear, monitoring of sports motions, the aerodynamics of football, and the injury prediction model. The typical parameters analyzed in this category are related to the kinematics and kinetics of the motion (e.g., peak linear and rotational acceleration, force-time curves, torques, angles, and velocity). The second macro-category involves the monitoring of physiological parameters; it includes monitoring of the training load and monitoring in the heat. The training load can be divided into internal load monitoring, which focuses on parameters describing the internal state of stress (e.g., HR, HRV, and the session rating of the perceived exertion) and the external load, which are parameters connected to the external stress (e.g., velocity/acceleration of running and player load). Monitoring in the heat instead focuses on monitoring the core temperature of the players and the time needed to reach exhaustion depending on the HR and %VO2. In Figure 5, we can observe how these two categories of studies developed throughout the years with increasing trends in the last five representing a general increase of interest in these topics.

### 3.2. Biomechanics of Concussion

A concussion is a severe injury with long-term outcomes, and the analysis of the biomechanics of concussions aims to limit, prevent, and understand the biomechanical causes behind their occurrence. With the objective of studying the biomechanics of concussions, the main research sub-categories include laboratory reconstruction (LAB) [49,50,51,52,53,54,55,56,57,58,59,60,61,62,63,64], monitoring with the head impact telemetry system (HIT) [65,66,67,68,69,70,71,72,73,74,75,76,77,78,79,80,81,82], wearable-sensor monitoring (WSM) [83,84,85,86,87,88,89,90,91], and computer modeling (CM) [92,93,94,95,96,97,98,99,100,101,102,103].

#### 3.2.1. Laboratory Reconstruction

Studies on LAB aimed to compute the severity of concussive and sub-concussive head impacts [49,60,61,62], evaluate the performances, or develop new helmet technologies [50,51,52,53,56,57,61,63], to develop new procedures, tools, and metrics for better simulations and reconstructions [54,55,58,59]. In Table 1, data regarding studies related to LAB are summarized.

The first experimental procedure on the biomechanics of concussion in professional American football athletes were based on video recordings of concussive or non-concussive impacts. These scenarios were then recreated in a laboratory using Hybrid III anthropomorphic test devices. The typical laboratory configuration was based on video analysis, which aimed to retrieve the initial kinematic description, including the impact location and velocity. The impact was then reconstructed using anthropomorphic test devices, which were instrumented with 9 accelerometers in a 3-2-2-2 configuration, capable of acquiring both linear and rotational accelerations.

Generally, the estimated errors ranged between 7% and 16% for linear acceleration and between 4% and 25% for rotational acceleration [49]. For this reason, Bailey et al. [55] proposed an optimized video analysis technique called videogrammetry, considering cameras with high frame rates. Videogrammetry was later employed in the reconstruction of helmet-to-helmet concussions [60] and helmet-to-ground concussions [61].

The study of helmet performance has been conducted at both the component level [50,53,63] and considering the helmet as a whole unit [52,56]. Specifically, researchers have analyzed the response curve of materials under stress at the component level [50,53] or the ability of new geometric designs of the helmet shell to minimize the measured peak linear acceleration [63].

Other papers analyzed current test protocols [71] or proposed new test procedures [54] and metrics [57,58,62] to evaluate helmet performance.

#### 3.2.2. Monitoring with Head Impact Telemetry System

In order to reduce the main issues related to LAB (such as time-consumption and a limited number of trials), wearable sensors were exploited (to collect large amounts of data in vivo during training and games). HIT is the most commonly used technology that is fit inside helmets; it is composed of spring-mounted accelerometers and telemetry hardware.

Studies on HIT collected data about the magnitude [65,66,67,68,69,70,71,73,74,75,76,77,78,79,81,87], frequency [72,73,75,79,80,81,82,84] and impact locations [67,76,78,81] of head impacts. In Table 2, data regarding studies related to HIT are summarized.

The magnitude of most experienced head impacts by players was skewed toward low severities, with a median peak linear acceleration of 20.5 g and a median peak rotational acceleration of 1400 rad/s${}^{2}$ [73].

For mild traumatic brain injury cases, the largest cohort using HIT with 51 cases reported a median peak linear acceleration of 66.7 g and a median peak rotational acceleration of 2963 rad/s${}^{2}$, whereas the largest cohort of LAB reported a mean peak linear acceleration of 72.2 g for concussions with loss of consciousness and 46.8 g for concussions without loss of consciousness. Moreover, the median peak rotational accelerations are 5247 rad/s${}^{2}$ concussions with loss of consciousness and 3457 rad/s${}^{2}$ for concussions without loss of consciousness. Concerning the frequency of impacts, in the cohort studied by Crisco et al. [72], the median number of seasons of head impacts experienced by a single player ranged from 257 to 438, and the maximum number of seasons of impacts ranged from 1022 to 1444 across 3 enrolled teams. The median impacts per game varied from 12.1 to 16.3, and the median impacts per practice varied from 4.8 to 6.6. In the largest reported cohort recently studied by Mccrea et al. [82], the median number of head impacts experienced by a player in a given season was 415 (interquartile range of 190–727), with the majority occurring during practices instead of games.

Regarding impact location, front impacts seem to be the most frequent [71,72] and are related to the highest peak rotational acceleration [76] and the lowest Gadd severity index [71]. Impacts to the top of the head are occurring at lower percentages and are related to higher peak linear acceleration [69,73,76] and the Gadd severity index [71]. Furthermore, the biomechanics of concussive and sub-concussive events is not associated with the clinical outcomes of concussions [67,68,75] or the trajectory of clinical recovery in terms of symptom resolution time and return-to-play time [81]. Sensory organization tests, automated neurophysiological assessments, and graded symptom checklists do not appear to be valid methods to discriminate between concussive and sub-concussive head impacts. Moreover, Harpham et al. [77] investigated the effect of visual performance on head impact severity and demonstrated how high performers in visual tests are less likely to be subjected to high-severity impacts, likely due to the higher level of awareness on the field.

#### 3.2.3. Wearable-Sensor Monitoring

Alternative wearable sensors are instrumented mouthguard sensors [83,85,86,87,88,90,91] and skin path sensors [89]. Regarding instrumented mouthguards, four papers dealt with the validation and development of instrumented mouthguard sensors [83,88,90,91], whereas the remaining papers from 2017 to 2019 [85,86,87] used mouthguard sensors to study and understand the relationship between the biomechanics of sub-concussive head impacts and variations in blood biomarkers. Two blood biomarkers, s100-beta [85] and neurofilament ligaments [87], were found to be related to head impact severity. A skin patch sensor was used in vivo to collect the biomechanical data about head impacts and to characterize different events, such as blocking, being blocked, tackling, being tackled, and ground contact [89]. In Table 3, data regarding studies related to HIT are summarized.

#### 3.2.4. Computer Modeling

LAB, HIT, and WSM are useful for providing descriptions of the kinematics of head impacts, but they cannot be used to extract measures of the consequences of impacts on the brain. Therefore, the consequences of concussion are usually evaluated by CM.

Studies on CM aimed to create new test protocols and metrics to screen the safety of equipment [92,93,97,102], develop and validate new tools and technologies [91,96,98,99], study the interaction between neck muscle activation and head impacts [95,100], and study the strain on the brain during practices and games [103] In Table 4, data regarding studies related to CM are summarized.

From 2013 to 2018, three articles [92,93,97] developed and validated a testing protocol involving a set of centric and non-centric impact locations justified by the different effects of the test configurations on the brain deformation metrics extracted by finite element modeling of the human brain. Finite element modeling applied to the brain was the most diffused modeling technique and it was also employed to compare concussive events with different clinical outcomes (loss of consciousness against non-loss of consciousness) [101].

Nevertheless, in recent years, finite element modeling research has expanded to equipment [98,99] and anthropomorphic devices [96], with good averages of similarities between the models, real-life helmets, and anthropomorphic devices. Additionally, computer modeling has been applied to models of neck muscle fibers and the skull during severe head impacts to understand the effect of muscle activation latency, muscle strength, and posture of the head-on injury metrics. Neck muscle strength does not seem to significantly affect head injury metrics [95,100], whereas early activation of neck muscles, representing an awareness of the impact [95], and a proper head posture [100], significantly decrease the injury metrics.

Finally, a recent paper [64] demonstrated how the same outcomes of brain deformations obtained with complex modeling of the brain can be achieved by convolutional neural networks, which take a map of the head kinematics as input.

### 3.3. Biomechanics of Foot-Wearing

Research on the footwear worn by American football players focuses on improving the design of both the shoe and the football field in order to reduce injury rates. Metatarsophalangeal joint sprain, also known as turf toe, is an injury mainly caused by hyperextension of the joint [104]. Therefore, footwear plays an important role in limiting the torque forces applied to this joint. Consequently, a line of research focuses on the quantification of forefoot bending stiffness (FBS) with the aim of understanding if the footwear is protective against metatarsophalangeal injuries [105,106,107]. On the other hand, a second line of research regards field–footwear interactions (FFI), taking into consideration the evidence of increased lower extremity injuries in artificial grass when compared to natural turf [108].

#### 3.3.1. Field–Footwear Interactions

The typical mechanism employed to simulate FFI is the Biocore Elite Athlete Shoe Turf [109,110,111], which is a machine that simulates a cleat moving on the turf. In 2015, a study by Kent et al. [109] quantified the differences in the mechanical interactions between artificial and natural surfaces using a cleated shoe used in both fields. The same methodological procedure was applied by Kent et al. [110], who employed 19 different kinds of cleated shoes on artificial and natural grass. In 2021, a study by Kent et al. [111] focused on the description of the mechanical response of the natural grass to the interaction with the cleated shoe. Only natural grass surfaces have inherent force-limiting qualities, which could explain the lower rates of injuries in these turf types [108]. On the contrary, on artificial turf, the footwear choice seems to be the most relevant characteristic to limit the occurrence of injuries. In Table 5, data regarding studies related to FFI are summarized.

#### 3.3.2. Footwear Bending Stiffness

Crandall et al. [105] and Lessley et al. [106] conducted dynamic testing to measure the torque and stiffness of various cleats, reporting range values of peak torques and peak stiffness relative to the flexion angle. Moreover, Crandall et al. [105] reported a high linear correlation (0.91) between shoe stiffness and peak torque. In a more recent study by Wannop et al. [107], ten American football players were enrolled to investigate the effect of three types of footwear with increasing bending stiffness while performing sport-specific movements. The forefoot bending stiffness of American football shoes does not produce enough torque to counteract the torques experienced by professional athletes [112] and, thus, the footwear is not protective against metatarsophalangeal hyperextension.

In Table 6, data regarding studies related to FBS are summarized.

### 3.4. Biomechanics of Sport-Related Movements

The biomechanics of sports-related movement (SM) employs a motion capture system comprising motion cameras and retroreflective markers to describe the kinematics and kinetics of the anatomical segments involved in specific sports actions. The common objective of these studies is to aid in the rehabilitation of athletes by providing medical staff with expected kinematics and kinetics in healthy individuals or to understand the mechanisms behind injuries. In Table 7, data regarding studies related to SM are summarized.

Rash and Shapiro [113] studied the biomechanics of throwing in twelve quarterbacks in their senior year of college. The throwing motion was analyzed using motion cameras and manual digitization of the frames. Riley et al. [114] published a motion analysis of the foot kinematics of nine American football players while they were performing three typical combined drills. The researchers used eight reflective markers on each foot and employed force plates to complete the description of the ground reaction forces. Deneweth et al. [115] reported position-specific hip and knee kinematics for forty NCAA athletes performing 45° cuts and side steps.

Among all player roles, linemen are at high risk of articular cartilage injuries [116,117], which motivates research on typical linemen drills. Lambach et al. [118] reported knee joint loading for fifteen linemen performing blocking drills and did not report any significant difference in knee compressive forces and moments between unloaded blocking drills and jogging or walking. They concluded that the blocking motion itself is not responsible for the elevated cartilage injury risk encountered in linemen. Future research should evaluate these movements in loaded conditions replicating game-like or practice-like conditions. Linemen also have the highest reported number of front-head impacts [72,73,79] compared to other roles. For this reason, Bonnechere et al. [119] analyzed the safest sprint starting position to reduce the occurrence and frequency of head impacts. Twelve American football players performed three sprints for three starting stances: 2-points (the two legs), 3-points (two legs and one arm), and 4-points (two legs, two arms) stances while wearing retroreflective markers.

**Table 7:** Studies regarding the biomechanics of SM.

	Study Design	Sample	Aim	Outcomes	QoA *
[113]	Prevalence descriptive study	12	Motion analysis of the football throw	average angular displacement in foot contact, maximum external rotation, release, angular velocities, forces, and torques	0.75
[114]	Prevalence descriptive stud	9	Describe ankle kinematics and the ground reaction forces in professional football players	patterns in the ground reaction forces, angular displacement curves, angular velocities curves	0.75
[115]	Prevalence descriptive study	40	analysis of hip and knee motion during game-like movements	hip and knee kinematics	0.75
[119]	Quasi-experimental	12	Understanding the safest stance position to prevent head impacts	trunk inclination, head inclination, verticality = (180-trunk inclination) + (180-trunk head); field of view = % height of head/verticality; redress time; head acceleration and velocity	0.89
[118]	Prevalence descriptive study	15	Motion analysis of the knee joint during linemen specific tasks	three-dimensional knee angles, joint reaction forces, external joint moments	0.75
[120]	Quasi-experimental	12	examination of the influence of high and low-cut footwear on the motion of athletes	tibial accelerations and three-dimensional kinematics of the lower body	0.78

* QoA stands for the quality of appraisal.

The authors computed trunk inclination, head inclination, and the field of view as a combination of the previous measures, head velocity, and acceleration along the vertical direction. From a motion analysis of the stance starts, subjects starting a sprint with a four-point stance and three-point stance are at a higher risk of head impacts due to the reduced field of view and increased vertical velocity of the head. These findings would explain why defensive linemen, usually starting with three points or four points, and offensive linemen, usually starting with three points, are subjected to a higher number of head impacts. They are just less aware of the head impact due to the limited field of view. In 2017, Sinclair et al. [120] evaluated the three-dimensional motion of the tibia in twelve players while they were running, changing direction, or jumping, and the authors studied the relationship of kinematic parameters to low-cut cleats and high-cut cleats. A proper choice of footwear influences the occurrence of injuries not only due to the interaction with the playing surface as stated beforehand but also due to its influence on tibia kinematics, with high-cut cleats providing greater medial support and limiting tibial acceleration, peak ankle joint eversion, and tibial internal rotations [120].

### 3.5. Aerodynamics of the Football and Catch

The aerodynamics of football and catch (AFC) aims at improving the performance of players by understanding what are the variables that can have an important effect of the trajectory of the football. The possible implications to performance improvement drives the interest of sports engineers; many studies have described the dynamics of rotating balls to better comprehend how the rotation of the ball can be modeled [121]. In Table 8, data regarding studies related to AFC are summarized.

Two papers that were selected studied the aerodynamics of the football during a kick. In 2016, Guzman, Brownell, and Kommer [122] studied and quantified the drag and lift coefficient of the football while rotating around its short axis in a wind channel. In 2018, Pfeifer et al. [123] simulated a kick with a machine to understand the optimal impact point and impact angle to maximize the distance. The researchers found that kicking the ball at 5.5 cm from the ground would yield the maximum distance with small insignificant variations, depending on the impact angle. Striking the ball at lower heights would instead produce higher launch angles and decrease the range.

Regarding the improvement of the catch, the authors of [124] proposed a prototype sensor to discriminate between catches and dropped balls.

### 3.6. Injury Prediction

The majority of the injuries experienced by football players are a consequence of the collisions occurring due to tackles and blocks, accounting for half of the total injuries in recent epidemiological investigations [15]. However, the occurrence of injuries without contact with other players represents the second most common mechanism [15] and the one that could be limited or avoided with prevention actions. Knees, shoulders, and ankles are the joints where athletes most often experience injuries, and this is why most of the research about injury prevention focuses on lower extremity injuries or shoulder injuries. Injury prediction (IP), which consists of creating mathematical models to recognize subjects at an elevated risk of injury and to identify possible predictors of future injuries during the season, is essential for injury prevention. In Table 9, data regarding studies related to IP are summarized.

Laudner [125] measured the level of shoulder instability in a cohort of 45 NCAA American football athletes compared to a control of 70 age-matched active people employing a force place to compute the radial area deviation of the center of pressure of an arm during a one-arm plank exercise. The authors showed a decreased sensorimotor control of the shoulder for the football cohort probably due to repetitive stress on the shoulder joint experienced by players due to tackling. A model for the prediction of shoulder injuries was proposed by Pontillo et al. [126]; it was fitted with the preseason data from 26 players. The preseason testing procedures were composed of questionnaire scores for upper extremity functions, range of motion screening, and the testing of shoulder fatigue.

A threshold of 21 touches in the closed-chain upper extremity stability test during the preseason achieved a sensitivity of 0.79 (95% confidence interval: 0.57–0.91) and a specificity of 0.83 (95% confidence interval: 0.44–0.97) in predicting shoulder injuries during the season [126].

Concerning core–lower extremities injuries, which represent the highest percentage of injuries occurring in American football [14,15], Wilkerson et al. [127] proposed a logistic regression 4-factor model fitted on data from 84 players in 2012, then refined it to a 3-factor model in 2015 [130] due to the greater number of players involved (n = 145 over 3 seasons). The subjects completed questionnaires during the preseason, including the Oswestry Disability Index, International Knee Documentation Committee, and components of the Foot and Ankle Ability Measure. Moreover, the authors administered tests to assess core endurance and aerobic capacity, monitoring recovery with the help of a HR sensor. Three factors were found to be the most discriminant for high injury risk established in preseason: the number of starting games higher than one, an Oswestry Disability Index score higher than four, and a wall-sit hold for less than 88 s. The presence of at least two of these three factors during the preseason corresponded to a sensitivity of 56% and a specificity of 80% [130]. The prediction of lower extremity injuries was also tackled with the lower quarter Y-balance test [128,132], consisting of the player standing on one leg and reaching (with the contralateral leg) the anterior and posterior directions, forming a Y shape. The results obtained by the authors when exploiting the lower quarter Y-balance test were positive for Butler et al. [128], showing 100% sensitivity and 71.7% specificity with a cut-off of 89.6% in the test for the prediction of in-season non-contact injury in the lower extremities. On the contrary, Luedke et al. [132] reported no significant differences in the scores between the uninjured and injured groups of players with a cohort composed of the same number of players, fifty-nine.

Honaker et al. [129] proposed a preseason test to discriminate between athletes with and without a history of concussion. Forty athletes, fifteen of whom had a history of concussion, participated in the study. The researchers used the gaze stabilization test, which assesses the vestibulo-ocular reflex’s ability to stabilize the gaze. The test measures the maximum head velocity in the yaw plane that a subject can perform while maintaining good vision. From this test, it is possible to measure the asymmetry score between right and left head rotations. A gaze stabilization asymmetry score higher than 13% achieved a sensitivity of 47% and a specificity of 96% [129], meaning that this test could be a valuable tool. However, due to the high number of false negatives, it should be used in combination with other tests to achieve better results. Another study on possible predictors of concussions with changes in variables measured before and after the season was proposed by Dubose et al. [131]. The authors analyzed a cohort of 39 players, 13 of whom experienced head trauma during the season. A motion capture system and a force plate were employed to record the kinematics and kinetics of the lower joints during a single stance stability test and jump test. The researchers noticed a statistically significant change in the stiffness of the lower extremities between baseline and post-season measurements for the concussed group, which could be interpreted as evidence of neuromuscular function changes.

### 3.7. Heat Monitoring of Physiological Parameters

Exertional heat illness can occur with various symptoms and severities, and it is possible to distinguish [133] between heat syncope (which happens to unfit or unacclimatized people in hot environments when standing for long periods of time or when rapidly changing posture), heat exhaustion (which represents an early cessation of exercise in hot environments due to multiple factors including cardiovascular strain, low blood pressure, and fatigue), exertional heat injury, and exertional heat stroke. Exertional heat illness is common in American football, with an average incidence rate of 1.31 per 1000 athlete exposures, ranging from 0.06 to 4.19 per 1000 athlete exposures across 7 studies analyzed in a recent systematic review [134]. Moreover, the same review reported American football as the field sport with the highest exertional heat illness incidence among other similar team sports. Heat monitoring (HM) aims to understand the contribution of the American football uniform to the exertional heat illness problem. In Table 10, data regarding studies related to HM are summarized.

Typical physiological parameters collected from subjects were the core temperature, measured from ingestible pills or a rectal thermistor [135,136,137,138,139,140,141,142], HR [135,136,137,139,140,141], skin temperature [137,138,140,142,143], and also measures of subjective perception taken from questionnaires, such as thirst scores, thermal sensation scores [135,137,138], and ratings of perceived exertion [135,136,137,138]. In Table 10, data regarding studies related to HM are summarized.

**Table 10:** Studies regarding HM.

	Study Design	Sample	Aim	Outcomes	QoA *
[135]	Sports science	15	Evaluation of the NCAA rule for the acclimatization of players	subjective perception: environmental subjective questionnaire, thirst and thermal sensations; physiological parameters: HR, temperature of the gastrointestinal tract	0.71
[136]	Randomized control trial	5	Evaluation of thermoregulatory, metabolic and cardiovascular responses	physiological parameters: HR, blood lactate, blood glucose, oxygen uptake, ratings of perceived exertion, core temperature	0.85
[137]	Randomized control trial	10	Evaluation of the effect of an American football uniform on the thermal response	subjective perception: Four scales of subjective perception were employed, i.e., a scale for thirst, a scale for thermal sensations, a rating of perceived exertion, and a scale for pain. Physiological parameters: rectal temperature with a rectal thermistor, forearm, and posterior neck skin temperatures, relative humidity under the jersey and T-shirt, HR, urine, and blood samples	0.85
[138]	Randomized control trial	10	Evaluation of the perceptual responses in the heat	subjective perception: environmental subjective questionnaire, ratings of perceived exertion, questionnaire for the thirst sensation, for muscle pain, for thermal sensation; physiological parameters: rectal temperature, temperature of the neck and forearm, time to reach 40 °C, internal uniform humidity	0.85
[143]	Sports science	14	heat loss estimation of linemen and non-linemen	physiological parameters: mean skin temperature as a weighted average of the chest, shoulder, quadriceps and calf; GPS-based measures of speed; measures of ambient conditions; indirect estimations of convective heat transfer coefficient, linear radiative heat transfer coefficient, combined heat transfer coefficient, sensible heat loss, evaporative heat transfer coefficient, maximum evaporative capacity, and maximum heat loss potential.	0.73
[139]	Sports science	13	Estimation of core temperature from wearable	physiological parameters: HR, core temperature; performance metrics: Lin’s concordance correlation coefficient, estimation bias	0.71
[142]	Sports science	15	Validity of body temperature sites for the evaluation of core temperature	physiological parameters: HR, temperatures measured at the center of the forehead, under the left armpit, under the tongue, and rectal temperature as ground truth	0.86
[141]	Randomized control trial	10	Using a heat tolerance test on athletes wearing pads and helmet	rectal temperature, HR, maximum oxygen uptake, and ratings of perceived exertion were taken during a maximal effort treadmill test at baseline	0.77
[140]	Quasi-experimental	5	Monitoring physiological index on different playing surfaces	physiological parameters: HR, breathing rate, energy expenditure, accelerometry score, sweat rate, core temperature, skin temperature on the neck, right shoulder, left hand, right shin, ratings of perceived exertion, local environmental conditions	0.78

* QoA stands for the quality of appraisal.

The research regarding heat monitoring started in 2006 [135] with the first studies on the heat response of athletes during preseason practices in the heat. From 2007 to 2010, three papers [136,137,138] simulated practice conditions and monitored the temperature of players with higher BMIs (linemen) because they were found to experience higher increments of temperature [135]. Moreover, linemen usually train at lower speeds than the other positions and experience lower levels of self-generated airspeed. As a consequence, it was concluded that linemen are at a higher risk of exertional heat illness due to their lower heat loss potential with convection and evaporation, which depend on the lower self-generated airspeed [143].

A recurring theme in papers on heat monitoring is how changes in worn equipment (e.g., wearing shoulder pads and helmets versus only wearing shorts) affect the temperature and perceived exertion of players. The American football uniform reduces heat dissipation, impairs thermoregulation [141], and consequently increases skin temperature and reduces the time to reach 40 °C [137,138]. For this reason, the NCAA acclimatization rule delaying the use of shoulder pads for the first few days of the preseason in the heat is supported by the higher rate of perceived exertion [136,137,138] reported by players, the higher energy cost expenditure measured by %VO2max [136] and the elevated core temperature [135] reported for the first few days of the preseason. Another aspect to consider in the acclimatization of the athletes is the playing surface. Artificial turfs have the lowest solar albedo and the highest temperature when compared to natural grass alternatives, leading to the greatest perceived heat for the athletes and the highest peak and average skin temperatures [140].

Finally, two recent papers [139,142] focused on monitoring the temperature and other physiological parameters of athletes in the heat. Common sites used to measure the temperature with skin patch sensors tend to underestimate the true core temperature recorded with rectal thermistors in players wearing American football uniforms [142]. As an alternative, an indirect estimation algorithm that takes heart rate as input was proposed [139].

### 3.8. Monitoring of the Training Load

Monitoring training loads (TLs) aims to improve athlete performance and reduce the risk of injuries. The use of wearable sensors provides a simple and effective way to track the workload experienced by athletes. Coaches can exploit the data extracted from wearables to tailor strength and conditioning programs to meet the specific needs of each player. Additionally, coaches can analyze the recorded data from practice and games to group athletes with similar needs. In Table 11, data regarding studies related to TLs are summarized.

Most of the papers [144,145,146,147,148,149,150,151,152,153,154,155,156,157,158,159,160,161,162,163,164,165,166,167,168] dealt with the topic of training loads in American football athletes and all of them measured external loads. Eight papers [147,153,154,159,164,165,166,168] measured internal load. Investigations of physical demands during practices were reported by five papers [144,151,157,163,167], whereas another five papers focused on game demands [145,148,150,158]. Furthermore, data extracted from accelerometers were used to assess the injury risks [146,155,161,162]. The common measure used in these types of studies is the acute-to-chronic ratio of player load, which is usually computed as the ratio between the player load in the week of the injury, labeled as acute workload, and the player load in the month of the injury (labeled as chronic workload). The monitoring of workload can assist coaches in the periodization of workload during the season or even during a single week [167]. The first week of the preseason appears to be the week with the highest workload compared to any other week of the season, including most of the game workload [151]. Wearables can be used to monitor the workload weekly and limit the occurrence of sudden increases in player load, which are associated with injury risk [127,155,161,162]. Another branch of research concerns the association between measures of external loads and measures of internal loads or subjective wellness. Wellness status is typically assessed through questionnaires that use a 0–5 scoring system for fatigue or energy, sleep quality, and muscle soreness [147,160], and in some cases, questionnaires may include sleep hours, mood, and stress levels [149,151]. Murray et al. [160] introduced the coefficient of multiple determination on the vertical direction of the accelerometer signal to determine the variability of the steps. Variability in the stride pattern, monitored through analysis of the vertical acceleration signal, can provide insights into how the player feels as it is associated with scores of subjective wellness [160].

**Table 11:** Studies regarding TL.

	Study Design	Sample	Aim	Outcomes	QoA *
[144]	Sports science	49	Evaluation of physical demands	external load: practice time, distance covered, maximal HR, average HR, percentage of covered distance and time in specific velocity zones; velocity categorized in zone 1 (standing: 0–1.0 km/h), zone 2 (walking: 1.1–6.0 km/h), zone 3 (jogging: 6.1–12.0 km/h), zone 4 (running: 12.1–16.0 km/h), and zone 5 (sprinting: more than 16.0 km/h)	0.82
[145]	Sports science	33	Evaluation of physical demands	external load: movements classified into low-intensity movements (0–10 km/h); moderate-intensity movements (10.1–16.0 km/h); high-intensity movements (16.1–23.0 km/h); and sprinting or maximal effort movements (exceeding 23.0 km/h). Movements classified by acceleration zones in moderate (1.5–2.5 m/s${}^{2}$), high (2.6–3.5 m/s${}^{2}$), and maximal (3.5 m/s${}^{2}$)	0.77
[146]	Sports science	45	Relationship between load and injury risk	external load: number of plays, average inertial load. Standard deviation of inertial load, coefficient of variation of inertial load	0.77
[147]	Sports science	58	Relationship between subjective wellness, player load and perceived exertion	internal load: session ratings of perceived exertion, a scale from 0 to 10, multiplied by the time of the training session; external load: player load; subjective wellness: ratings from 1 to 5 for three questionnaire items being muscle soreness, sleep and energy	0.68
[148]	Sports science	40	Quantification of average and maximum distances traveled in games	external load:total distance, low (0 to 12.9 km/h), moderate (12.9 to 22.5 km/h), moderate-high (>19.3 km/h), high (>22.5 km/h) intensity distance; max range = computed as the range from the mean distance +1SD to max distance	0.82
[150]	Sports science	33	Examination of positional impact profile	external load: accelerometer data divided into an impact classification system of 6 zones: 5 to 6 m/s${}^{2}$ (very light), 6.1 to 6.5 m/s${}^{2}$ (light to moderate), 6.6 to 7.0 m/s${}^{2}$ (moderate to heavy), 7.1 to 8.0 m/s${}^{2}$ (heavy impact), 8.1 to 10.0 m/s${}^{2}$ (very heavy impact), higher than 10 m/s${}^{2}$ (Severe impact)	0.91
[151]	Sports science	31	Evaluation of physical demands	external load: player load, peak player load, average player load, cumulative player load	0.96
[149]	Sports science	32	Evaluation of physical demands	external load: player load, total, low intensity, medium intensity, high intensity, sprint running distances (m), acceleration distance, and deceleration distance. Movements classified into low- (0 to 12.9 km/h), medium- (13 to 19.3 km/h), high- (19.4 to 25.8 km/h), and maximal- (≥25.9 km/h) intensity efforts. Classification of acceleration and deceleration motions in low (0 to 1 m/s${}^{2}$), medium (1.1 to 2.0 m/s${}^{2}$), high (2.1 to 3.0 m/s${}^{2}$), and maximal (higher than 3 m/s${}^{2}$); subjective wellness: questionnaire scale of 1 to 5 for fatigue, sleep quality, soreness, stress, mood, and hours of sleep	0.82
[152]	Sports science	30	Relationship between perceived wellness and load	same as [149]	0.86
[159]	Sports science	29	Monitoring cardiac autonomic activity	internal load: natural logarithm root mean square of successive differences; resting heart rate; external load: player load	0.81
[153]	Sports science	-	Monitoring cardiac autonomic activity	internal load: natural logarithm root mean square of successive differences; resting heart rate; external load: player load	0.81
[155]	Sports science	52	Relationship between load and injury risk	external load: player load; acute workload for each week of the season. Acute-to-chronic ratios were computed relative to injuries within 3-day or 7-day lag periods and computed as the ratio between 7/14, 7/21, and 7/28 using an exponentially weighted moving average	0.86
[156]	Sports science	40	Quantification of workloads	external load: player load; low- (1.5–2.5 m/s), moderate- (2.5–3.5 m/s), and high-intensity (>3.5 m/s) accelerations, decelerations, and left or right change of direction. Total movement workload	0.73
[157]	Sports science	63	Quantification of workloads between different positions	external load: total distance, high-speed running distance equal to distance with speed above 70% threshold of max speed computed from the previous year’s observations. Player load, player load per min, inertial movement analysis	0.86
[158]	Sports science	43	Monitoring physical demands	external load: Distance traveled, maximum velocity, total inertial movement analysis, acceleration/deceleration data clustered in category	0.68
[154]	Case report	1	Case report of HRV-monitoring for a concussive case	internal load: natural logarithm root mean square of successive differences; resting heart rate external load: player load	0.86
[160]	Sports science	63	Relationships between load, wellness, soreness, and stride variability	external load: player load, acute-to-chronic ratio, coefficient of multiple determination evaluated on the step waveforms extracted from the vertical direction of the accelerometer signals; subjective wellness: questionnaire for fatigue, sleep quality, and muscle soreness.	0.82
[161]	Sports science	42	Relationship between wellness score, acute-to-chronic ratio, and injury risk	external load: acute-to-chronic ratio; subjective wellness: wellness questionnaire for soreness, energy, and sleep quality	0.82
[162]	Sports science	232	Relationship between player workload and soft tissue injuries	external load: acute-to-chronic ratio; subjective wellness: wellness questionnaire for soreness, energy, and sleep quality	0.77
[163]	Sports science	66	Clustering workload	external load: max velocity, inertial movement analysis, player load, distance ran at 5 to 8 mph, distance ran at 8 to 12 mph, distance ran at 12 to 16 mph, and distance ran at 16 to 25 mph; number of snaps	0.77
[168]	Sports science	30	Relationships between internal and external load	internal load: session ratings of perceived exertion, HR zones in 0–60% and 60–70% and 70–80% and 80–90% and 90–95%, total HR exertion, training impulse, maximum HR, average HR, HR load, energy expenditure, recovery; external load: distances covered in speed zones-standing and walking (0 to 6 km/h), jogging (6–12 km/h), cruising (12–14 km/h), striding (14–18 km/h), high-intensity running (18–20 km/h), and sprinting (>20 km/h); data were divided also in low-intensity distance (0–14 km/h) and high-intensity distance(>14 km/h); max speed, number of sprints (>20 km/h) and total distance. Acceleration classified into four categories: 0.5 to 0.99, 1 to 1.99, 2 to 2.99 and >3	0.82
[164]	Sports science	23	Evaluation of physical demands	internal load: average HR, max HR, time to peak HR; external load: mean activity, integrated activity	0.91
[165]	Sports science	-	Monitoring HRV throughout a season	internal load: natural logarithm root mean square of successive differences; resting heart rate; external load: player load	0.95
[166]	Sports science	17	Relationship between training load and next-day recovery	internal load: physiological load is a heart rate-based metrics from 0 to 10 where 0 corresponds to 50% of age-predicted heart rate and 10 to 100% of max age-predicted HR; s-RPE x time practice; external load: mechanical load given by the peak acceleration along any direction scaled from 0 (0.5 g) to 10 (>6 g); recovery status: reactive strength index test, perceived restorativeness scale questionnaire	0.82
[167]	Sports science	72	Evaluation of physical demands	external load: high-speed running per min, sprint distance per min (distances covered above the 12 mph and 15 mph), player load, inertial movement analysis	0.68

* QoA stands for the quality of appraisal.

In four of the analyzed papers [144,164,166,168], the internal load measures were extracted from wearable sensors. Another four papers published by Flatt et al., from 2018 to 2021 [153,154,159,165], opened up a new research line regarding the monitoring of HRV and cardiovascular recovery between training days in American football athletes. The HRV was sampled thanks to mobile devices and finger sensors just before each training session; the subjects wore GPS sensors to collect player load data. The metric used to understand the effect of the autonomic nervous system on cardiovascular function was a time-domain index of vagal tone. The researchers documented the behavior of HRV during spring camp [153], in-season practices [159], and across the entire season from preseason to post-season [165], stratifying the results by player position. Flatt et al. [154] reported on the daily fluctuations of HRV experienced by a concussed player. Monitoring internal load is crucial to understand whether players have the appropriate recovery time. It has been shown that linemen experience a decrease in vagal tone throughout the season [153,154,165], and they do not fully recover baseline vagal activity on consecutive training days [159]. This parasympathetic impairment observed in linemen is due to multiple contributing factors, such as a progressive increase in physiological stress as the season progresses, a high frequency of soft tissue traumas, and a high frequency of sub-concussive impacts [165]. Monitoring HRV could provide coaches with the necessary information to better plan practices throughout the week, ensuring adequate recovery time between them.

## 4. Discussion

American football is a sport that attracts different branches of the biomedical engineering field. Due to this increasing interest, the aim of the present scoping review was to report the applications of biomedical engineering research in the sport of American football, highlighting the main topics and possible challenges. The literature search was conducted systematically, following the PRISMA guidelines [38] and the extended guidelines for scoping reviews [39]. Given the choice of keywords in the literature search and the exclusion criteria, we could have removed some important papers or topics. The two biggest removed clusters regarded studies on young populations (individuals under 18 years of age) and studies that focused on screening procedures, as well as those that investigated strength training, plyometrics training, and combined drills.

Given its high injury rates, the biomechanical analysis of athletes is a primary area of research aimed at evaluating musculoskeletal injuries and concussions. This research also includes an interest in the design of helmets and shoulder pads as preventative tools. Advances in wearable and portable sensors have enabled researchers to perform tests and acquire data directly in the field, providing information on biomechanical events, external workload, and internal workload. These practices allow for monitoring of both the health status of players and their performance during training sessions and competitions.

It was found that the most important branches of research on the studied topic were biomechanical studies regarding injuries encompassing concussions [49,50,51,52,53,54,55,56,57,58,59,60,61,62,63,64,65,66,67,68,69,70,71,72,73,74,75,76,77,78,79,80,81,82,83,84,85,86,87,88,89,90,91,92,93,94,95,96,97,98,99,100,101,102,103], footwear [105,106,107,109,110,111], and sports-specific motion [113,114,115,118,119,120]. The most researched line is related to the biomechanics of concussions. The reasons for this are likely related to the connection between concussion and the dangerous consequences associated with cases of mild head traumatic injury in the long term [19]. An issue that emerges from the literature is the fact that concussion was shown to be a complex multidimensional event that does not depend solely on severity but also on impact location, frequency of impacts, posture of the head, neck muscle activation patterns [95,100], and visual performance of the athletes [77]. Considering the contribution of many different factors, a player could experience a mild traumatic brain injury of varying severity, and different players could have differing clinical recovery patterns [67,68,75,81]. Thus, a universal concussion threshold based solely on measures of severity is difficult to define. Despite that, the recent technology of wearable and portable sensors allows the collection of large databases, which can be used to create optimized risk curves, which is reliable for population screening [74].

Head telemetry systems and mouthguard sensors should be the focus of future research, considering their ability to record large datasets and the drawbacks of time-consuming and error-prone laboratory reconstructions [49]. Data-driven models, such as neural network architectures, could provide new objective and automatic methods to discriminate between concussive and sub-concussive events. The issue related to the identification of sub-concussive events remains an open problem in the literature. Additionally, the description of concussion occurrence by only linear acceleration is limited [51,71,102]. The single use of metrics (such as the Gadd severity index or head injury criterion) is not adequate because they are only associated with linear acceleration, whereas rotational acceleration seems to play an essential role in the identification of concussions [93,97]. Measurements of brain deformation, such as maximal principal strain and von Mises stress, have been shown to characterize the performance of helmets [92] and should be added to the protocols of performance evaluation.

One recent proposed test protocol [57,58,62] seems promising because it includes a new metric, i.e., a head acceleration response metric based on a linear combination of both linear (e.g., head injury criterion) and rotational (e.g., diffuse axonal multi-axis general evaluation) acceleration metrics. To complete the characterization of a concussion event, metrics should include measures of brain strain and deformations that could be indirectly computed from the kinematics [64]. Regarding the prevention and limitation of concussions, a great percentage of concussions and overall exposure to head impacts occur during practices instead of competitions. To reduce this trend, a new regulation was applied in the NCAA in 2019, eliminating two-a-day practices, but it was unsuccessful [80]. The easiest way to tackle the problem of concussion is to act directly in practice by reducing the amount of contact and overall head impact exposure. In this scenario, coaches and institutions have a pivotal role in promoting changes and the use of wearable sensors to continuously monitor athletes.

Collecting information through wearable sensors, which is already widespread in concussion monitoring, is lacking in the field of footwear applications and the biomechanics of sports motion. One possible drawback of the analyzed literature is that all the results regarding the mechanical interactions between the footwear and playing field were reported in simulated environments with a machine replicating the force and weight of an athlete. Wearables could solve this issue, and foot-mounted or shoe-mounted wearable devices are already available to provide valuable information regarding running gait mechanics [169,170]. Therefore, these sensors could also be used for sprinting, changing directions, blocking actions, and other sport-specific motion analyses. Moreover, the use of retroreflective markers in conjunction with motion cameras has been the major technique used to evaluate the biomechanics of sport-specific movements. Thus, contactless methods based on video analysis or radar analysis could be promising to extract important information without the need for wearing sensors. Future industrial research in the field of footwear should focus on improving the innovation of artificial turf design, with the aim of reproducing the mechanical qualities of natural grass [111], and the design of shoes, with the aim of finding a trade-off between performance, comfort, and prevention [105,106,107]. Despite many studies evaluating the design of helmets, no studies dealt with the evaluation of new designs of shoulder pads; only one study proposed an integrated solution of helmet and shoulder pads [56]. Shoulder pads protect against shoulder, neck, and chest injuries; therefore, it could be interesting to expand research to the biomechanics of other equipment.

The second largest branch of research connected with American football involves the monitoring of physiological parameters, workloads, and performances. The main applications are the monitoring of external and internal loads [144,145,146,147,148,149,150,151,152,153,154,155,156,157,158,159,160,161,162,163,164,165,166,167,168], monitoring of temperature [135,136,137,138,139,140,141,142,143] and catch rates [124]. Heat monitoring was performed in most of the analyses using invasive methods. However, the literature shows that the inaccuracy of skin patch sensors is a major problem [142]. Thus, future research should focus on investigating new non-invasive ways to extract temperature data from athletes during practice and competition, given the fatal outcome of exertional heat illness. Regarding load monitoring, all of the evaluated literature dealt with external workloads, but eight of the analyzed papers [147,153,154,159,164,165,166,168] collected data on internal loads and used wearables to extract heart rate series [164,166,168], four of which used portable sensors to monitor heart rate variability [153,154,159,165], and one considered a perceived exertion questionnaire [147]. Future research should focus on expanding the use of wearable sensors to monitor heart rate and heart rate variability. Moreover, given the novel technology based on portable devices, the application of portable sensors for electrocardiogram recordings [171,172] and electromyogram recordings [173,174] should be investigated. Another issue found in the analyzed literature was the lack of investigation into respiration rates in real scenarios. However, given the importance of this vital sign [175] in the context of load, fatigue, and stress monitoring, future research could focus on investigating respiration monitoring through wearable sensors during training sessions and games.

Regarding performance monitoring, an innovative approach [124] was to evaluate the catch rate of players during training sessions using a prototype of wearable sensors worn on the palms of players’ hands. The study of football kicks [122,123] focused on the aerodynamics of the football and optimizing the impact location and angle of the kick. In this context, future research could involve the use of motion cameras, depth cameras, or wearable sensors to study movements or improve the technical capability of the player kicking the ball. Alternatively, other contactless methods could include using micro-Doppler signatures from radar sensors to distinguish between different sport functional movements [176] or using it as a screening tool for injury prevention to recognize possible abnormalities in simple walking mechanisms [177,178].

Finally, studies related to the prediction of injuries [125,126,127,128,129,130,131,132] looked at the best predictors of in-season injuries between multiple tests at baseline during preseasons. The results for different kinds of injuries (from lower body and core injuries to shoulder injuries and concussions) were promising, despite being tested on small sample sizes without internal validation, which led to contradictory results [128,132].

## 5. Conclusions

In conclusion, this scoping review evaluated the trends in the biomedical engineering of American football, highlighting the fields with the most research and the fields lacking research lines. In this sport, the prevalence of injuries and the consequences of these injuries are the main topics of research, including the biomechanics of concussions, heat monitoring, and injury prevention. Player safety is surely the most common objective that is leading to innovation in helmet and footwear design or research into the mechanisms of injury occurrence. However, no studies were found that dealt with the development, validation, and innovation of shoulder pads, which are the main sources of protection against shoulder and neck injuries. Additionally, coaches and players are interested in improving performances on the field, which has motivated all of the studies regarding workload monitoring. However, internal load monitoring is limited. A large gap in the literature on American football involves the monitoring of the vital signs of athletes. A complete description of the internal load statuses of athletes via heart rate and respiration rate during practice and competition should be further investigated.

## Figures and Tables

**Table 1:** Studies regarding the biomechanics of concussions studied by LAB.

	Study Design	Sample	Aim	Outcomes	QoA *
[49]	Case-control	31	Limitations and errors of LAB	magnitude: peak linear and rotational acceleration; impact location; impact kinematics	0.62
[50]	Simulation	120	Testing performance of a helmet subcomponent	measure of performance of a shock absorber	0.85
ine [51]	Simulation	195	Comparison of performance between helmets	magnitude: peak linear and rotational acceleration; angular velocity; injury metrics: the Gadd severity index	0.83
ine [52]	Simulation	1600	Evaluation of helmet performance	linear acceleration response curves	0.77
[53]	Simulation	120	Testing performance of a helmet subcomponent	peak force, time to peak force, peak temperature, step change temperatures, tensile modulus, yield stress, ultimate tensile stress	0.75
[54]	Simulation	24	Development of a test protocol for helmet performance	magnitude: peak linear acceleration; injury metrics: the Gadd severity index, head injury criterion	0.71
[55]	Simulation	10	Evaluation of the videogrammetry technique	pre- and post-impact kinematics	0.83
[56]	Simulation	96	Evaluation of performance of a new tech	magnitude: peak linear and rotation acceleration; injury metrics: the Gadd severity index, head injury criterion	0.81
[57]	Simulation	1116	Evaluation of helmet performance	injury metrics: head acceleration response metric, diffuse axonal multi-axis general evaluation, head injury criterion, helmet performance score	0.79
[58]	Simulation	1512	Development of a metric for helmet performance	same as [57]	0.89
[59]	Case series	57	Videogrammetry	impact location; changes in velocities, impact velocity, change in rotational velocity vector component, closing velocities	0.78
[60]	Case-control	100	Videogrammetry	magnitude: peak linear and rotational acceleration; impact location; closing velocity; composite input and output kinematics error score; injury metrics: head injury criterion, diffuse axonal multi-axis general evaluation	0.88
[61]	Case-control	16	Videogrammetry	initial kinematics, linear velocity changes, angular velocity changes, the ratio between linear velocity change and horizontal linear velocity, the ratio between angular velocity change and initial angular velocity	0.75
[62]	Simulation	56	Evaluation of the shell products for linemen	performance metrics: same as [57]	0.85
[63]	Simulation	27	Evaluation of folding patterns geometries for new helmet design	magnitude: peak linear acceleration; a performance score computed as a weighted average of peak linear acceleration values for three tested velocities	0.79
[64]	Simulation	1104	Estimation of strain measures through neural network	deformation: peak maximal principal strain and peak cumulative damage strain measure	0.81

* QoA stands for the quality of appraisal.

**Table 2:** Studies regarding the biomechanics of concussions studied by HIT.

	Study Design	Sample	Aim	Outcomes	QoA *
[65]	Sports science	38	Monitoring head impacts	magnitude: peak linear and rotational acceleration; injury metrics: the Gadd severity index, head injury criterion	0.71
[66]	Sports science	38	Monitoring head impacts	magnitude: peak linear and rotational acceleration	0.67
[67]	pre-post observational no control	88	How severity affects the outcome of concussion	magnitude: peak linear and rotational acceleration; impact location; scores for symptoms, balance, memory; concussion history	0.7
[68]	Prospective longitudinal cohort	43	How severity affects the brain functions	magnitude: peak linear acceleration; scores for symptoms, balance, and memory	0.73
[69]	Sports science	72	Monitoring of head impacts	magnitude: peak linear acceleration; injury metrics: the Gadd severity index, head injury criterion	0.76
[70]	Sports science	10	Monitoring head impacts	magnitude: peak linear acceleration; injury metrics: the Gadd severity index	0.67
[71]	Sports science	40	Evaluation of a test protocol	magnitude: peak linear and rotational acceleration; injury metrics: the Gadd severity index, head injury criterion	0.71
[72]	Sports science	188	Monitoring head impacts	frequency: total impacts in season, practice, game and impacts per practice, game	0.67
[73]	Sports science	314	Monitoring head impacts	magnitude: peak linear and rotational acceleration, head impact severity; frequency: same as [72]	0.67
[74]	Sports science	98	Development of concussion risk curve	magnitude: peak linear acceleration; concussion risk curve; injury metric: head injury criterion	High Bias
[75]	Before–after study with no control	46	How severity affects brain impairment	magnitude: total cumulative magnitude of impacts; frequency: total number of impacts, total number of impacts greater than 90 g, total impacts to the top of the helmet; concussion history, years in college football, sensory organization test, graded symptoms checklist	0.7
[76]	Sports science	254	Monitoring head impacts	magnitude: peak linear and rotational acceleration, head impact severity; impact location	0.67
[77]	Before–after study with no control	38	Relationship between visual or sensory performance and severity	magnitude: peak linear and rotational acceleration, head impact severity	0.64
[78]	Sports science	33	Monitoring of head impact location	magnitude: peak linear and rotational acceleration; impact location; injury metrics: the Gadd severity index, head injury criterion	0.89
[79]	Sports science	340	Monitoring of head impacts	magnitude: peak linear and rotational acceleration; frequency: total number of head impacts during practice, the number of head impacts per practice	0.76
[80]	Sports science	342	Monitoring of head impacts after elimination of 2-a-day practices	frequency: head impacts per week and per day, total number of head impacts, contact intensity defined as the number of head impacts per day; number of contact practice days, number of contact practice sessions, duration of contact practice sessions, number of two-a-day practice sessions	0.76
[81]	Before–after study with no control	45	Relationship between concussion biomechanics and symptoms, clinical recovery, and return-to-play	magnitude: peak linear and rotational acceleration; frequency: season and injury day repetitive head impact exposure, computed as the number of impacts sustained by the players in the considered period; impact location; symptom severity score, error score from balance error scoring system, score from standardized assessment of concussion; complete symptom resolution time, return-to-play time	0.8
[82]	Sports science	658	Investigation of head impact exposure during one season	frequency: head impact exposure as the number of impacts in games and practices	0.81

* QoA stands for the quality of appraisal.

**Table 3:** Studies regarding the biomechanics of concussions studied by WSM.

	Study Design	Sample	Aim	Outcomes	QoA *
[83]	Simulation	5	Evaluation of a novel-instrumented mouthguard	magnitude: peak linear and rotational acceleration; impact location; angular velocity	0.83
[84]	Sports science	16	Monitoring head impacts	magnitude: cumulative impact load per event and season, peak linear and rotational acceleration; frequency: number of hits per practice type, number of impacts over a threshold of peak linear and rotational acceleration	0.81
[85]	Before–after study with no control	22	Plasma S100-beta as a biomarker of subconcussive hits	magnitude: peak linear and rotational acceleration; frequency: number of hits; s100-beta concentration, symptoms score	0.73
[86]	Before–after study with no control	23	Plasma Tau as a biomarker of subconcussive hits	magnitude: peak linear and rotational acceleration; frequency: number of hits; s100-beta concentration; Tau concentration; symptom score; near point of convergence	0.8
[87]	Before–after study with no control	18	Plasma neurofilament light chain as a biomarker of concussive hits	magnitude: peak linear and rotational acceleration; frequency: number of hits; s100-beta concentration; Tau concentration; neurofilament light chain concentration; symptom score; near point of convergence	0.8
[88]	Sports science	21	Development, evaluation of mouthguard with integrated machine learning for head impacts detection	magnitude: peak linear and rotational acceleration; angular velocity; features of pulse size, power spectral density measures and kinematic-based measures; injury metrics: head injury criterion, diffuse axonal multi-axis general evaluation	0.76
[89]	Sports science	7	Comparison between head kinematics of different contact events	magnitude: peak linear and rotational acceleration, peak angular velocity	0.52
[90]	Simulation	60	Comparison between head kinematics of different contact events	magnitude: peak linear and rotational acceleration, peak angular velocity	0.77
[91]	Sports science	18	Development of a mouthguard sensor	peaks of head kinematics, peak occurrence times; deformation: 95% maximal principal strain, 95% maximal principal strain rate; relative error in each truncated kinematic case	0.57

* QoA stands for the quality of appraisal.

**Table 4:** Studies regarding the biomechanics of concussion studied by CM.

	Study Design	Sample	Aim	Outcomes	QoA *
[92]	Simulation	27	Development of a new test protocol	magnitude: peak linear and rotational acceleration, peak angular velocity; deformation: maximal principal strain, von Mises stress	0.85
[93]	Simulation	81	Proposal of an impact protocol	magnitude: peak linear and rotational acceleration, peak angular velocity; deformation: maximal principal strain, von Mises stress	0.85
[94]	Case series	2	Estimation of the Brain Injury	magnitude: peak linear and rotational acceleration, peak angular velocity; deformation: strain, strain rate, von Mises stress; injury metrics: the Gadd severity index, head injury criterion, rotational injury criterion, generalized acceleration model for brain injury threshold, brain injury criterion	0.56
[95]	Simulation	4	Relationship between neck muscles and concussion risk	magnitude: peak linear and rotational acceleration, peak angular velocity; deformation: maximal principal strain, maximum shear strain, cumulative strain damage measure; injury metrics: head injury criterion, brain injury criterion, peak intracranial pressure	0.83
[96]	Simulation	42	Development and validation of finite element models of Hybrid III head/neck and impactor	rating metrics to compute similarity; acceleration-time curves	0.79
[97]	Simulation	32	Evaluation of impact site and impact type on the concussion risk	magnitude: peak linear and rotational acceleration, peak angular velocity; deformation: maximal principal strain, von Mises stress	0.77
[98]	Simulation	35	Bottom-up approach for a finite element football helmet	finite element model	0.83
[99]	Simulation	97	Development and evaluation of a finite element model of a new helmet	measures of similarity: correlational analysis and composite correlation analysis	0.83
[100]	Simulation	2880	Relationship between neck muscles and concussion risk	skull kinematics; injury metrics: head impact criterion, brain injury criterion, head impact power	0.83
[101]	Case-control	-	Comparison between concussions with and without loss of consciousness	magnitude: peak linear and rotational acceleration, peak angular velocity; deformation: maximal principal strain, above cumulative strain damage measure 10%, strain rate; pre-impact kinematics: velocity at which the impact occurred, impact location;	0.78
[102]	Simulation	8	Comparison between intracranial pressure and head injury criterion during linear impact tests	injury metrics: intracranial pressure, head injury criterion	0.83
[103]	Prevalence study	168	Evaluation of brain deformations for different roles	deformation: strain rate, maximal principal strain; impact location, impact velocity, event type, linear and rotational velocity/acceleration;	0.75

* QoA stands for the quality of appraisal.

**Table 5:** Studies regarding the biomechanics of FFI.

	Study Design	Sample	Aim	Outcomes	QoA *
[109]	Simulation	24	Quantification of the mechanical interaction between American football cleats and surfaces	peaks of forces and torques; displacement-time curves, rotation-time curves	0.79
[110]	Simulation	57	Quantification of the mechanical interaction between different cleats and surfaces in conditions similar to play	linear regression analysis to find relationships between horizontal forces during the translation tests and the torques	0.79
[111]	Simulation	15	Quantification of peak load in natural grass and define the load–displacement response	force-time curves; load–displacement corridors	0.83

* QoA stands for the quality of appraisal.

**Table 6:** Studies regarding the biomechanics of FBS.

	Study Design	Sample	Aim	Outcomes	QoA *
[105]	Simulation	21	Quantification of the forefoot bending stiffness in American football footwear	torque and stiffness	0.83
[106]	Simulation	30	Quantification of the forefoot bending stiffness of American football shoes	torque and stiffness; flexion scores	0.81
[107]	Quasi-experimental	10	Effect of forefoot stiffness on the metatarsophalangeal joint extension and athletic performance	maximal metatarsophalangeal extension	0.78

* QoA stands for the quality of appraisal.

**Table 8:** Studies regarding AFC.

	Study Design	Sample	Aim	Outcomes	QoA *
[122]	Simulation	-	Evaluation of the aerodynamics of the football using a wind chamber	drag and lift forces and coefficients	0.62
[123]	Simulation	-	Evaluation of the best location to kick the football for the distance and height	trajectory, total distance	0.69
[124]	Sports science	8	Monitoring the catch rate with a convolutional neural network	magnetometer, audio, and pressure signals were used as input to classify catch and non-catch events	0.76

* QoA stands for the quality of appraisal.

**Table 9:** Studies regarding IP.

	Study Design	Sample	Aim	Outcomes	QoA *
[125]	Quasi-experimental	59	Analysis of the upper extremity sensorimotor control in American football players	radial area deviation; receiver operating characteristic analysis	0.78
[126]	Prediction model, Longitudinal cohort	26	Prediction of shoulder injury from preseason variables	closed kinetic chain upper extremity stability test: start in a plank position, bring one hand over the other and then come back to the original position and repeat with the opposite hand. Goal = the highest number of touches in 15 s; sensitivity and specificity	High Bias
[127]	Prediction model, Longitudinal cohort	83	Prediction of core and lower extremity injuries from preseason test variables	model features; questionnaires: Oswestry disability index, international knee documentation committee, sports component of the foot and ankle ability measure; core endurance tests: horizontal back-extension hold, sitting 60° trunk flexion holds, side-bridge holds, bilateral wall-sit holds. Aerobic capacity test: 3-min step test. physiological index: HR with polar telemetry to assess recovery; multiple linear regression analysis and receiver operating characteristic analysis	High Bias
[128]	Prediction model, Longitudinal cohort	59	Prediction of lower extremities injuries with a functional balance test at preseason	Lower Quarter Y-Balance Test test score	High Bias
[129]	Prediction model, Longitudinal cohort	40	Evaluation of the gaze stabilization asymmetry score as a screening tool for concussion	stability evaluation test, gaze stabilization test, dizziness handicap inventory score; receiver operating characteristic analysis, sensitivity and specificity	High Bias
[130]	Prediction model, Longitudinal cohort	152	Refined prediction of core and lower extremity injuries from preseason test variables	model features: questionnaires: Oswestry disability index, international knee documentation committee, sports component of the foot and ankle ability measure; core endurance tests: horizontal back-extension hold, sitting 60° trunk flexion holds, side-bridge holds, bilateral wall-sit holds. Aerobic capacity test: 3-min step test. Physiological index: HR with polar telemetry to assess recovery; multiple linear regression analysis and receiver operating characteristic analysis	High Bias
[131]	Prediction model, longitudinal cohort	39	Measure of change in stiffness in the lower extremities from pre to post season as an indicator of concussion	force, kinematics, moments, peak flexion angle, peak external flexion moments. Joint stiffness	High Bias
[132]	Prediction model, Longitudinal cohort	59	Prediction of lower extremities injuries with a functional balance test at preseason	Lower Quarter Y-Balance Test test score	High Bias

* QoA stands for the quality of appraisal.

## Data Availability

Not applicable.

## Abbreviations

The following abbreviations are used in this manuscript:

AFC	Aerodynamics of the Football and Catch
BMI	body mass index
CM	computer modeling
DOAJ	Directory of Open Access Journals
FBS	footwear bending stiffness
FFI	field–footwear interaction
GPS	global positioning system
HIT	head impact telemetry system
HM	heat monitoring
HR	heart rate
HRV	heart rate variability
IP	injury prediction
LAB	laboratory reconstructions
NCAA	National Collegiate Athletic Association
PRISMA	preferred reporting items for systematic reviews and meta-analyses
QoA	quality of appraisal
SM	sport-related movement
TL	training load
WSM	wearable-sensor monitoring
